# Vindel: a simple pipeline for checking indel redundancy

**DOI:** 10.1186/s12859-014-0359-1

**Published:** 2014-11-19

**Authors:** Zhiyi Li, Xiaowei Wu, Bin He, Liqing Zhang

**Affiliations:** Department of Computer Science, Virginia Tech, Blacksburg, VA 24061 USA; Department of Statistics, Virginia Tech, Blacksburg, VA 24061 USA

**Keywords:** Indel redundancy, Gap opening, Gap extension

## Abstract

**Background:**

With the advance of next generation sequencing (NGS) technologies, a large number of insertion and deletion (indel) variants have been identified in human populations. Despite much research into variant calling, it has been found that a non-negligible proportion of the identified indel variants might be false positives due to sequencing errors, artifacts caused by ambiguous alignments, and annotation errors.

**Results:**

In this paper, we examine indel redundancy in dbSNP, one of the central databases for indel variants, and develop a standalone computational pipeline, dubbed Vindel, to detect redundant indels. The pipeline first applies indel position information to form candidate redundant groups, then performs indel mutations to the reference genome to generate corresponding indel variant substrings. Finally the indel variant substrings in the same candidate redundant groups are compared in a pairwise fashion to identify redundant indels. We applied our pipeline to check for redundancy in the human indels in dbSNP. Our pipeline identified approximately 8% redundancy in insertion type indels, 12% in deletion type indels, and overall 10% for insertions and deletions combined. These numbers are largely consistent across all human autosomes. We also investigated indel size distribution and adjacent indel distance distribution for a better understanding of the mechanisms generating indel variants.

**Conclusions:**

Vindel, a simple yet effective computational pipeline, can be used to check whether a set of indels are redundant with respect to those already in the database of interest such as NCBI’s dbSNP. Of the approximately 5.9 million indels we examined, nearly 0.6 million are redundant, revealing a serious limitation in the current indel annotation. Statistics results prove the consistency of the pipeline on indel redundancy detection for all 22 chromosomes. Apart from the standalone Vindel pipeline, the indel redundancy check algorithm is also implemented in the web server http://bioinformatics.cs.vt.edu/zhanglab/indelRedundant.php.

**Electronic supplementary material:**

The online version of this article (doi:10.1186/s12859-014-0359-1) contains supplementary material, which is available to authorized users.

## Background

Genetic variations include single nucleotide polymorphisms (SNPs), insertions and deletions (indels), and structural variants such as inversions, large-scale duplications/deletions, and transpositions. Among all these types of variation, indels are the second most common in human populations, only after SNPs, demonstrated by recent large-scale human genome sequencing projects [1]^i^. However, with the availability of newly sequenced human genomes, the number of novel indels increases at a much faster pace than that of SNPs. For example, a 2011 study shows that more than 63% of the nearly 2 million indels identified in 79 diverse human genomes are novel [2], compared to those in dbSNP. Recently, sequencing and analysis of an Indian female’s genome reveals that about 84% of her indels are unique, i.e., not documented in any of the sequenced genome databases, in contrast to less than 3% of her SNPs being unique [3]. Thus, compared to SNPs, the research on cataloging indel variants is still in its infancy, and intense effort is needed to obtain a complete inventory. Indels also present great technical difficulty and challenge to short read mapping algorithms. Improved from the first generation short read mappers, various mapping programs and indel detection programs have been developed to allow for indel detection [4-12]. However, if indels happen to occur in seed regions (where only mismatches are allowed), mappers and indel detection programs may still fail to map the reads, though it is unclear how this impacts the overall mapping performance.

**Figure 1:**
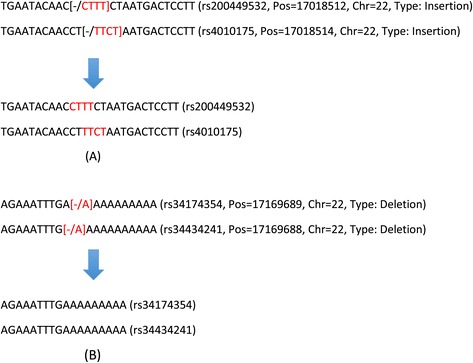
**Examples of indel redundancy in dbSNP. (A)** Two indels, both of insertion type, result in the same variant sequences with respect to the reference sequence **(B)** Two indels, both of deletion type, result in the same variant sequences with respect to the reference sequence.

**Figure 2:**
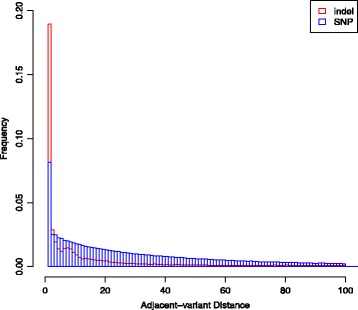
**Histograms of adjacent-SNP distances and adjacent-indel distances (before redundancy filtration) on human chromosome 22.** Histograms are plotted in probability densities, with blue color representing SNPs and red representing indels.

With rapid development in indel-related research, quality evaluation of the identified indels becomes more and more important to downstream association studies. Compared to SNP calling, indel calling is more prone to errors occurring in PCR, sequencing, mapping, and calling procedures. These errors can lead to a high false positive rate in indel identification [13]. On the other hand, due to much stringent criteria and highly involved process of indel calling, real indels could also be removed [14]. Recently, while working on indels in dbSNP, we noticed that multiple indels result in the same mutation but are treated as different variants. Figure 1 shows examples of such indels with one insertion type and one deletion type. For both types, the two indels are presented as different variants in the current version of dbSNP with distinct IDs, but sequence alignments show clearly that they cause the same change to the reference genome. Biologically, indels with alternative positions (as seen in the current example) may exist, but to our knowledge, experimentally, there is no way of knowing exactly which one represents the true biological signal. As will be shown later, there exist a non-negligible number of such redundant indels in the current dbSNP. Note we call indels redundant if they differ only in annotations, not in the resulting sequences after their modifications to the reference genome. This redundant information does not reveal real biological signals and may mislead downstream analyses. The observed redundancy could be due to equally optimal sequence alignments produced by alignment programs, that is, when the variant sequence and the reference sequence are aligned together, alignment programs cannot differentiate multiple optimal solutions computationally, hence different indel variants may be reported by different alignment programs. These cases suggest that redundancy might be a general problem for indels curated in dbSNP. However, to our knowledge, no work has been done to fully explore the extent of redundancy. To get further confirmation, we compare the distance distribution of adjacent SNPs with that of adjacent indels in dbSNP. It is found that in all chromosomes, compared to the distances of adjacent SNPs, the distances of adjacent indels show a higher proportion at distance one. As an illustrative example, Figure 2 shows histograms of the distances between adjacent SNPs and the distances between adjacent indels for chromosome 22. Both types of distances have monotone decreasing distribution. However, in sharp contrast to SNPs, the number of adjacent-indel distance =1 stands out from all other distances. This shows that compared to SNPs, there are many more indels that are right next to each other or are very close on a chromosome. This further motivates us to examine the redundancy in indels, especially among nearby indels.

This paper develops methods and strategies to check for indel redundancy. Using dbSNP indels as the test case, we examine the extent of indel redundancy for humans and develop Vindel, a standalone indel redundancy verification pipeline, together with a corresponding web tool. Statistics analysis is applied to check for the correctness of the pipeline. As indels have been shown to be linked to diseases and cancer and have been used as genetic markers for various purposes, it is essential to catalog redundant indels and develop annotations with non-redundant information that represent real biological signals instead of computational artifacts. Our Vindel system provides the tool needed for this purpose.

## Methods

Based on the two examples of redundant indels in dbSNP (Figure 1) and the comparison of distance distribution of adjacent SNPs and indels (Figure 2 for chromosome 22, see Additional file 1: Figure S1 for other chromosomes), we designed and implemented an indel redundancy verification pipeline. The pipeline consists of three phases: First, indel information was retrieved from the SNP/indel flat files downloaded from dbSNP^ii^ using a Python program. Second, based on their position information, indels were allocated into candidate redundant indel groups by clustering. Third, indel variant substrings were generated correspondingly for indels in the same candidate redundant groups and pairwise comparisons were conducted to identify redundant indels. Details are described in the following.

### Data retrieval

NCBI dbSNP is a widely used public database for short genetic variants. We collected indel information by parsing the human genome dbSNP (GRCh37 build version p10) flat files that contain both SNPs and indels for chromosomes 1 to 22. The original files were parsed to retrieve indel information, including indel ID, chromosome number, chromosome position, allele information, and alignment type.

### Indel alignment type specification verification

To check indel redundancy, we need to determine the alignment type (insertion or deletion) for each indel relative to the reference genome. NCBI dbSNP^iii^ specifies indel alignment types in four categories, with loctype = 1 denoting “insertion on the subject sequence”, Loctype = 3 “deletion on the subject sequence”, Loctype = 4 “range insertion”, and Loctype = 6 “range deletion”. In this work, we focus on small indels, i.e., loctype = 1 and loctype = 3, which account for the majority of human indels (>99%). As it is unclear from the description page what the subject sequence means in the annotation and our email inquiry to dbSNP helpdesk was not answered, we checked the reference genome to see whether the substrings exist, so the type corresponds to deletion of the substrings. Our results show that indels with loctype 1 should correspond to deletion made to the reference genome as only about 1% of loctype 1 failed to find the substring versus more than 38% of loctype 3. Therefore, from here on, we treat loctype 1 as deletion and loctype 3 as insertion relative to the reference genome.

### Candidate redundant indel groups

Based on the retrieved indel position information, we scanned indels along each chromosome to form candidate redundant indel groups. To limit the number of pairwise comparisons in the subsequent step, we apply further filtrations to the indels when generating candidate redundant indel groups: (1) Because indels belonging to different types (insertion and deletion) or having different lengths are definitely not the same variant, we only consider indels with the same type and length; (2) We also set a threshold value for the distance between adjacent indels of the same group since redundancy is less likely to happen between two indels located far away from each other. Indels were then grouped into candidate redundant groups if their distances are less than the threshold value. Each candidate redundant indel group may contain two or more indels. Details are shown in Table 1.

**Table 1:** **Algorithm for clustering indels into candidate redundant indel groups**

**Algorithm 1:**	Clustering indels into candidate redundant indel groups Algorithm
**Input:**	An indel List: *List* (*I*) ordered by indel positions on the reference genome, each indel *I* has position *P*, threshold value *D* of distance between adjacent indels
**Output:**	Candidate redundant indel groups List *List* (*G*_*k*_)
1	Candidate-Group-Generation (indel list: *List*(*I*), threshold-value: *D*)
2	Set List (*G*_*k*_) empty: Ø;
3	Set *k =* 0;
4	Set current indel *I*_*current*_ = *I*_*0*_, the first element in the *List* (*I*);
5	**for** each indel *i* = *2* to *n* in indel list *List* (*I*)
6	**if** next adjacent indel *I*_*i*_’s position *P*_*i*_ - *P*_*current*_ < = *D* **then**
7	Add the next indel into the current candidate group *G *(*k*);
8	Set current indel *I*_*current*_ *= I*_*i*_;
9	**else**
10	Append *G(k)* to candidate group list *List *(*G*_*k*_);
11	*k = k* + *1*;
12	**end**
13	**return** candidate redundant group list *List* (*G*_*k*_);

### Indel redundancy check

**Figure 3:**
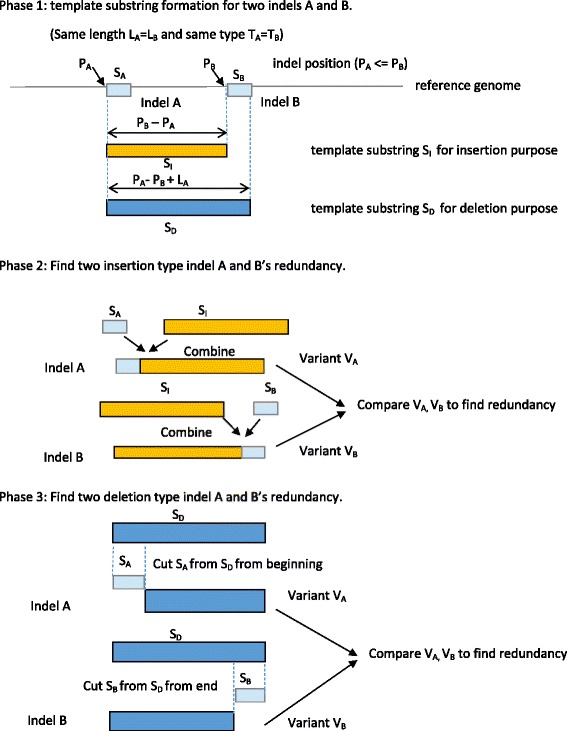
**A demonstration of how we check whether two indel variants are the same.**

To find redundancy in candidate redundant indel groups, we conducted pairwise comparison for indel variant substrings generated from indels in the same group. For each indel, we generated the variant substring based on indel position, allele information, alignment types, and the corresponding reference genome substring. The human reference genome, GRCh37 build version p10, is downloaded from NCBI^iv^. Consider two indels A and B in a candidate group, and without loss of generality, assume that A precedes B on the chromosome. If both A and B are of insertion type, we extract a template substring from A’s position to B’s position on the corresponding reference genome (Figure 3), attach indel A to the front of the template to form A’s variant string, and attach indel B to the end of the template to form indel B’s variant string. These two variant strings are then compared for equivalence, in other words, if the two variant strings are identical, then one indel is redundant with respect to the other. Similarly, if both indels A and B are of deletion type, we extract a template substring from indel A’s starting position to B’s ending position on the reference chromosome, delete indel A from the beginning to form indel A variant, and delete indel B from the end to form indel B variant. The comparison between the two variant substrings then determines whether redundancy exists between the two indels. The algorithm details are shown in Table 2.

**Table 2:** **Algorithm for indel pair redundancy check by applying sliding window on reference genome**

**Algorithm 2:**	Indel pair redundancy check Algorithm
**Input:**	Two candidate redundant indels *A* and *B*’s information
	same type *T* (either insertion or deletion), *T*_*A*_ = *T*_*B*,_, same length *L*_*A*_ = *L*_*B*,_
	allele information *S*_*A*,_ *S*_*B*,_ position information *P*_*A*,_ *P*_*B*,_ where *P*_*A*_ < = *P*_*B*,_
	reference genome sequence *S*.
**Output:**	A pair indels *A*, *B* are redundant or not: Redundancy
1	Set Redundancy = *False*;
2	Phase 1: template substring formation
3	Form template substring for insertion type *S*_*I*_ or for deletion type *S*_*D*_ separately
4	*S*_*I*_ *=* Substring in reference genome with *P*_*B*_ - *P*_*A*_;
5	*S*_*D*_ = Substring in reference genome with *P*_*B*_ - *P*_*A*_ + *L*_*A*_;
6	Phase 2: variant substring formation for insertion type
7	**if** *T*_*A*_ = *T*_*B*_ = Insertion **then**
8	Insert *S*_*A*_ in front of template substring *S*_*I*_ to form variant substring *V*_*A*_ for indel *A*;
9	Append *S*_*B*_ at the end of template substring *S*_*I*_ to form variant substring *V*_*B*_ for indel *B*;
10	**if** *V*_*A*_ = *V*_*B*_ **then**
11	Redundancy found: Redundancy = *True*;
12	**else**
13	No Redundancy
14	Phase 3: variant substring formation for deletion type
15	**if** *T*_*A*_ = *T*_*B*_ = Deletion **then**
16	Cut *S*_*A*_ in front of template string *S*_*D*_, form variant substring *V*_*A*_ for indel *A*;
17	Cut *S*_*B*_ at the end of template string *S*_*D*_, form variant substring *V*_*B*_ for indel *B*;
18	**if** *V*_*A*_ = *V*_*B*_ **then**
19	Redundancy found: Redundancy = *True*;
20	**else**
21	No Redundancy
22	**return** Redundancy

### Statistical analysis of the distribution of indel sizes and adjacent-indel distances

**Figure 4:**
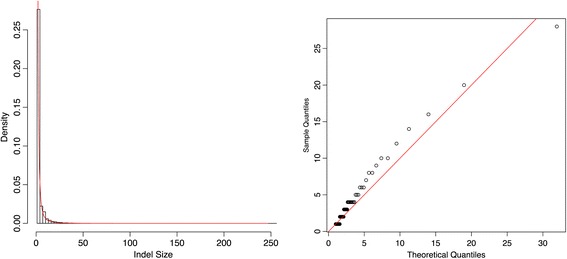
**Fitting Pareto distribution to indel sizes for chromosome 22.** Left panel: indel size histogram with fitted Pareto density function shown in red line; Right panel: QQ-plot (sample quantiles of indel sizes vs. quantiles of the fitted Pareto distribution).

We first consider the size of indels after removing redundant indels. It has been shown that the distribution of indel sizes in human and rodent pseudogenes can be described well by a power law distribution [15,16]. In our study, we perform a similar analysis. As an example, Figure 4 shows the result of fitting a Pareto distribution to the indel sizes on chromosome 22. We set the location or scale parameter of the Pareto to be 1 since the minimum possible value of the indel sizes is 1. By maximum likelihood estimation (MLE), the estimated shape parameter is $\widehat{\alpha}=1.33$.

**Figure 5:**
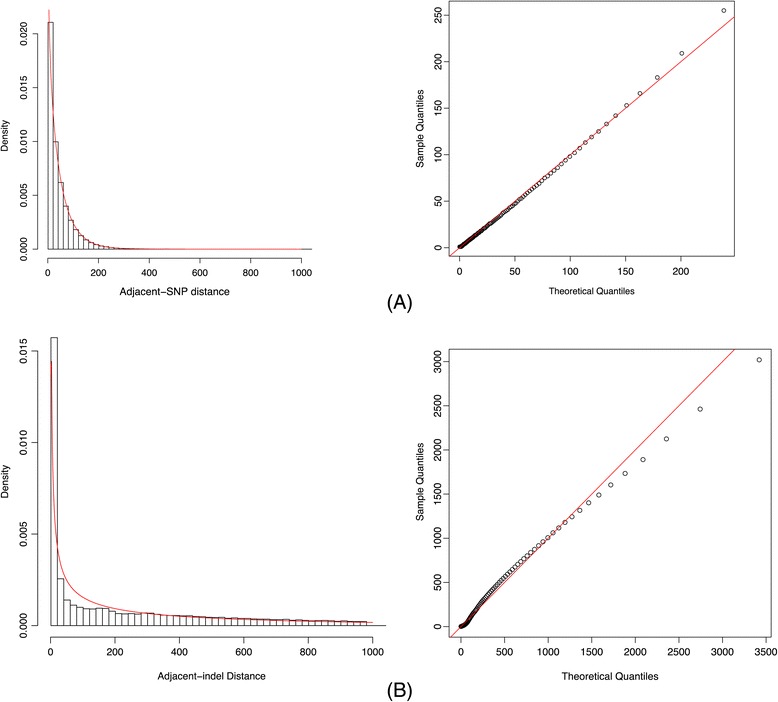
**Fitting gamma distribution to adjacent-SNP distances and adjacent-indel distances for chromosome 22. (A)** Fitting gamma distribution to adjacent-SNP distances. Left panel: fitted gamma density function shown in red, observed distribution in black; Right Panel: adjacent-SNP distance QQ-plot (sample quantiles vs. quantiles of the fitted gamma distribution). **(B)** Fitting gamma distribution to adjacent-indel distances (after redundancy filtration). Left panel: adjacent-indel distance histogram with fitted gamma density function shown in red line; Right Panel: adjacent-indel distance QQ-plot (sample quantiles vs. quantiles of the fitted gamma distribution).

**Figure 6:**
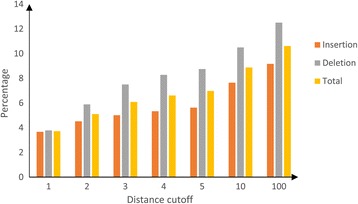
**The percentage of redundant indels as a function of distance threshold for human chromosome 22.** Orange column represents Insertion type indels; Gray column represents Deletion type indels; Yellow column represents total inels (Insertion type + Deletion type).

Next, we consider modeling the distribution of distances between adjacent indels. We take the distance between adjacent SNPs as an illustrative example. The histogram in Figure 2 suggests that the distance between adjacent SNPs may be approximated by an exponential distribution, which implies that the occurrence of SNPs in the human genome may follow a Poisson process. To add flexibility (i.e., allow for more dispersion) in modeling, here we use a Gamma distribution to fit the distribution of distances between adjacent SNPs. The corresponding count data model, often called the Gamma count model, is a renewal process with Gamma inter-arrivals [17,18]. This model is widely used in the analysis of genetic applications, for example, modeling the occurrence of gene conversion [19] and the occurrence of crossovers [20]. When the shape parameter α of the Gamma distribution is taken to be an integer, this model can be naturally thought of as counting every α^th^ event as an arrival in a Poisson process. As an example, Figure 5 shows the result of fitting a Gamma distribution to the distribution of distances between adjacent SNPs on chromosome 22, with the MLE of shape parameter $\widehat{\alpha}=0.83$ and the MLE of the rate parameter $\widehat{\beta}=0.018$. Alternatively, if we fit the distance distribution of adjacent variants with an exponential distribution, the MLE of the rate parameter $\widehat{\lambda}=0.021$. We note that these two distributions have similar means and variances, and the log likelihoods for observing such adjacent-SNP distances under the Gamma and exponential distributions are − 3.589 × 10^6^ and − 3.599 × 10^6^, respectively. Analogously, we fit a Gamma distribution to the distribution of distances between adjacent indels after redundancy filtration. Figure 2 suggests that great over-dispersion should be observed for the distribution of distances between adjacent indels in comparison to that of adjacent SNPs. In other words, the rate parameter is expected to be much smaller for the adjacent-indel distance distribution than that for the adjacent-SNP distance distribution. Figure 6 demonstrates an example of fitting a Gamma distribution to the adjacent-indel distances on chromosome 22. The resulting estimated parameters are $\widehat{\alpha}=0.39$ and $\widehat{\beta}=8.77\times {10}^{-4},$ and the log likelihood is − 5.22 × 10^5^. Other distributions such as Weibull may also be used to model the adjacent-indel distance distribution in observing its heavy tail. However, the goodness-of-fit may not be better than the Gamma count model. In the example of chromosome 22, the log likelihood under Weibull (with MLE: shape parameter 0.52, scale parameter 257.43) distribution is − 5.23 × 10^5^.

## Results and discussion

### Indel redundancy rate with different distance cutoffs

We first applied our pipeline to human indels on chromosome 22. As pairwise comparison of all the indels on a chromosome is too time consuming, we set cutoff values for the distance between adjacent indels to 1, 5, 10, and 100 bps. We generated candidate redundant indel groups based on these cutoff values, then applied indel redundancy verification methods to identify redundant indels. In this process, we calculated the redundancy percentage for indel insertion type and deletion type separately since we only handled alignment type 1 (Deletion) and 3 (Insertion) as discussed in the method section. The results are shown in Figure 6. As the distance threshold increases from 1 to 5, the total indel redundancy rate increases sharply, from 3.72% to 8.88%, however, from 10 to 100, the percentage increase trend becomes flat, from 8.88% to 10.62%, suggesting that for large distance groups, there is less increase in the number of redundant indels. Specifically, for distance threshold 100, we get 13% redundancy rate for insertion type indels and 9% redundancy rate for deletion type indels. Based on this observation, we adopted the cutoff of 100 as the distance cutoff for identifying redundant indels on all chromosomes.

### Indel redundancy rates for all the chromosomes

**Figure 7:**
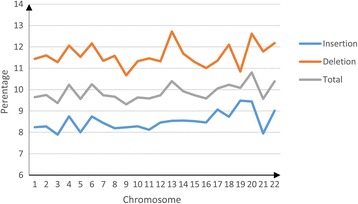
**The percentage of redundant indels for human chromosome across 1–22.** Blue line represents Insertion type indels; Orange line represents Deletion type indels; Gray line represents total inels (Insertion type + Deletion type).

We applied our redundancy check pipeline to indels from chromosome 1 to chromosome 22 to see how redundancy rate varies with chromosome. Figure 7 shows that the redundancy rate for insertion type indels, deletion type indels, and total indels. On average, 9.77% of the total indels are identified as redundant. The redundancy rate is quite consistent across chromosomes (Table 3). The standard deviation of these redundancy rates is 0.35%. Such a small variation in redundancy rate implies that redundancy occurs homogeneously among all 22 chromosomes and indel redundancy problem is not biased towards any particular chromosome. We also provide the proportion of insertions over redundant indels, as shown in Table 3. It is found that the numbers of redundant insertions and deletions are comparable among all 22 chromosomes, though on average, there are fewer insertions(proportion: 49%) shown in redundant indels than deletions. The slight discrepancy between the rates of redundancy for insertions and deletions may be due to the more frequent occurrence of deletions than insertions.

**Table 3:** **Various statistics**

**Chromosome**	**Original number of indels**	**Redundancy**	**Insertion redundancy**	**Indel size**	**Adjacent-SNP**		**Adjacent-indel**	
		**rate (%)** ^**a**^	**rate (%)** ^**b**^	**Shape** ^**c**^	**Shape** ^**d**^	**Rate** ^**e**^	**Shape** ^**f**^	**Rate** ^**g**^
1	485270	9.63	47.99	1.44	0.83	0.016	0.39	7.55E-04
2	503609	9.57	48.18	1.43	0.86	0.016	0.4	7.57E-04
3	431820	9.38	47.57	1.47	0.89	0.017	0.4	8.03E-04
4	417942	9.98	48.62	1.47	0.89	0.017	0.39	7.82E-04
5	374774	9.49	47.06	1.46	0.88	0.017	0.4	7.53E-04
6	388741	10.16	48.14	1.43	0.84	0.017	0.39	7.99E-04
7	349376	9.68	48.38	1.43	0.86	0.017	0.4	7.96E-04
8	307212	9.55	48.56	1.47	0.88	0.018	0.4	7.72E-04
9	250802	9.29	49.25	1.45	0.82	0.016	0.4	7.37E-04
10	292264	9.58	48.23	1.42	0.85	0.017	0.39	7.82E-04
11	280673	9.6	47.66	1.46	0.87	0.017	0.4	7.72E-04
12	298606	9.65	48.73	1.45	0.87	0.017	0.4	8.16E-04
13	223181	10.31	46.21	1.39	0.9	0.017	0.38	7.95E-04
14	195779	9.8	49.23	1.46	0.88	0.017	0.4	7.94E-04
15	182417	9.77	48.87	1.44	0.85	0.016	0.4	7.86E-04
16	180020	9.41	50.29	1.39	0.81	0.018	0.4	8.09E-04
17	185888	10.03	51.8	1.4	0.83	0.016	0.4	8.40E-04
18	169830	10.02	48.61	1.41	0.89	0.017	0.4	8.07E-04
19	148904	9.93	53.31	1.39	0.81	0.018	0.41	9.69E-04
20	139927	10.54	51.3	1.4	0.88	0.018	0.39	8.16E-04
21	96577	9.3	49.52	1.45	0.85	0.018	0.4	9.83E-04
22	90621	10.31	50.46	1.33	0.83	0.018	0.39	8.77E-04
Mean	5994233^h^	9.77	49	1.43	0.86	0.017	0.4	8.09E-04
STD		0.35	1.62	0.035	2.73E-02	7.61E-04	5.54E-03	6.21E-05

^a^The total redundancy rate on individual chromosomes. ^b^The percentage of redundant insertions. ^c^The shape parameter of the Pareto distributions fitted to the indel sizes (after redundancy filtration). ^d,e^The shape and rate parameter estimates for the Gamma distributions fitted to the adjacent-SNP distances. ^f,g^The shape and rate parameter estimates for the Gamma distributions fitted to the adjacent-indel distances. ^h^Total number of indels studied.

**Figure 8:**
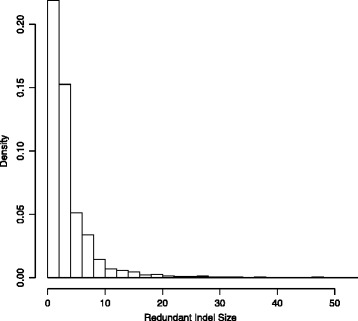
**Histogram of redundant indel sizes for human chromosome 22.**

In addition, it can also be seen that the size of redundant indels follows a particular distribution. Intuitively, redundancy tends to occur more frequently between indels with small sizes. This can be seen clearly from the histogram of redundant indel sizes on chromosome 22 (Figure 8).

### Indel size distribution

After redundancy filtration, we fit the indel size distribution with a Pareto distribution. The scale or location parameter is fixed to be 1. MLE of the shape parameters for all 22 chromosomes are listed in Table 3. The shape parameter varies little across chromosomes, ranging from 1.33 to 1.47, with mean 1.43 and standard deviation 0.035. This is consistent with the shape parameter of [15,16].

### Distribution of distances between adjacent variants

We first investigate the distribution of distances between adjacent SNPs. By fitting the distance distribution with a Gamma distribution, we obtain MLE for the shape parameter α and rate parameter β for all 22 chromosomes (see Table 3). The shape parameter estimates are all close to (though smaller than) 1, suggesting that the occurrence of SNPs on human chromosomes may be described approximately by a Poisson process with rate 0.017 *times* the genetic distance within humans. On the other hand, after removing the redundant indels, we fit the distribution of distances between adjacent indels with a Gamma distribution. The parameter estimates for all 22 chromosomes are listed in Table 3, with average $\widehat{\alpha}=0.4$, and average $\widehat{\beta}=8.09\times {10}^{-4}$. Comparing the corresponding parameter estimates $\widehat{\alpha}$ and $\widehat{\beta}$ in the Gamma count models for the distance distribution of adjacent SNPs and for the distance distribution of adjacent indels, we see that the mean adjacent indel distance is $\frac{0.4/8.09\times {10}^{-4}}{0.86/0.017}=9.77$ times larger than the mean adjacent SNP distance, and also the variance is $\frac{0.4/{8.09}^2\times {10}^{-8}}{0.86/{0.017}^2}=205.38\sqrt{2}$ times larger. This result indicates several differences between SNPs and indels. First, the rate of indel mutations might be about ten times lower than that of single nucleotide mutations. Large-scale genome sequencing projects [1,21,22] have shown that there are about 1 SNP every 100 bps, whereas about 1 indel per 1000 bps. The rate difference could also be partially contributed by the fact that indels are under stronger selective constraints than SNPs and stronger purifying selection on indels might have removed more indels than SNPs.

Several aspects merit discussion. First, results on modeling indel size distribution and modeling adjacent-indel distance distribution can be used to estimate gap extension and gap opening in sequence alignment and indel calling algorithms. Second, as we limited the distance cutoff to be 100, there is still redundancy, albeit small, in indels that are farther apart than 100 bps. Therefore, future improvement includes incorporation of more efficient algorithm for examining all possible indel pairs to identify all the redundant indels. Furthermore, other important features, such as sequencing errors, mapping errors, and coverage, may also be incorporated in our algorithm to aid the selection of distance cutoff. One may argue that sequence alignment ambiguity may also reflect true biological events, in the sense that there are correspondingly multiple ways for indels to happen. However, if we focus on the net effect of these variations, it is clear that regardless of the exact indel events, they create the same variant string or genomic sequence and therefore, should most likely have the same effect on the individual carrying the variant. Therefore, we believe that it is important to keep only the unique indels.

### A web-based tool for indel redundancy check process based on standalone pipeline algorithm

In addition of the standalone indel redundancy check pipeline, we also developed a Web-based user friendly tool for indel redundancy check. The Web tool applies PHP/APACHE/MYSQL/Linux architecture, based on a Model-View-Controller design strategy. In the Web interface front end, a user can input indel information, such as chromosome number, chromosome position, and indel allele information. In the server backend, we have a database table that stores indel information from dbSNPs and the indel redundancy check pipeline Python program that checks for redundancy based on the user’s input. The redundancy checking results are displayed in the Web front end. For computational efficiency, the current Web tool only searches for and checks adjacent indels less than 5 bps from the user’s query indel in our non-redundant indel database. The response time is at most a few seconds and the result is displayed to the Web front end. However, for their target indel, users can also choose to examine all indels on the same chromosome as the target indel for redundancy check, which significantly increases the computational time. One limitation with the current redundancy check standalone pipeline and Web tool is that they only handle indels in humans. As more and more indels from other species are identified, we will add the capability of indel redundancy check for additional species.

## Conclusions

Based on the observed indel redundancy in the current dbSNP, we developed Vindel, a simple computational pipeline to check for indel redundancy in a database of interest. Vindel is implemented in Python and used to investigate the degree of redundancy in human indels in dbSNP. The approximate 10% redundancy is observed consistently across all the 22 human chromosomes. Further statistics results prove the consistency of the pipeline. In addition to the standalone Vindel pipeline, the indel redundancy check algorithm is also implemented in the Web server http://bioinformatics.cs.vt.edu/zhanglab/indelRedundant.php.

### Endnotes

^i^http://massgenomics.org/2012/01/the-current-state-of-dbsnp.html.

^ii^http://www.ncbi.nlm.nih.gov/SNP/.

^iii^http://www.ncbi.nlm.nih.gov/SNP/specs/alignment_types.html.

^iv^ftp://ftp.ncbi.nlm.nih.gov/genbank/genomes/Eukaryotes/vetebrates_mammals/Homo_sapiens/GRCh37/Primary_Assembly/assembled/chromosomes/FASTA

## Additional file

Additional file 1: **Supplementary materials for Vindel: a simple pipeline for checking indel redundancy.** This additional file includes histograms of indels and SNPs for Chromosome 1–22, and statistics analysis results for Chromosome 1–22.
