# Supplementary material for: Vindel: a simple pipeline for checking indel redundancy
Source: BMC Bioinformatics. 2014 Nov 19;15(1):359. doi: 10.1186/s12859-014-0359-1 (PMC4245841; doi:10.1186/s12859-014-0359-1)
Supplement: Supplementary file 1 — Supplementary materials for Vindel: a simple pipeline for checking indel redundancy. This additional file includes histograms of indels and SNPs for Chromosome 1–22, and statistics analysis results for Chromosome 1–22. [file 12859_2014_359_MOESM1_ESM.docx]

**Additional file 1: Supplementary materials for**

**Vindel: a simple pipeline for checking indel redundancy**

Zhiyi Li^1,3^, Xiaowei Wu^2,3^, Bin He^1^, Liqing Zhang^1,4^

^1^ Department of Computer Science, Virginia Tech. Blacksburg, VA 24060

^2^ Department of Statistics, Virginia Tech. Blacksburg, VA 24060

^3^ These two authors contributed equally to the paper.

^4^ Corresponding author.

Figure 1. Histograms of adjacent-SNP distances and adjacent-indel distances (before redundancy filtration) for human chromosome 1-22 (data is from dbSNP GRCh37 build version p10)


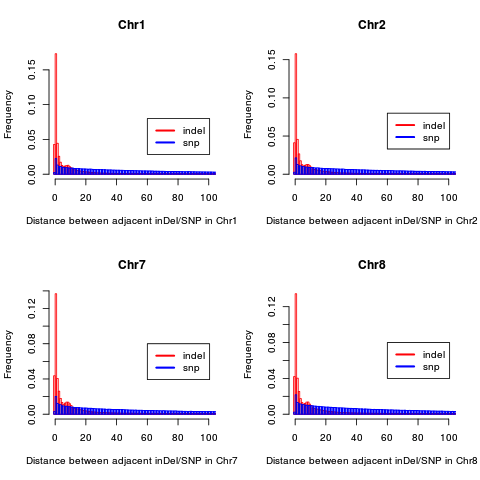

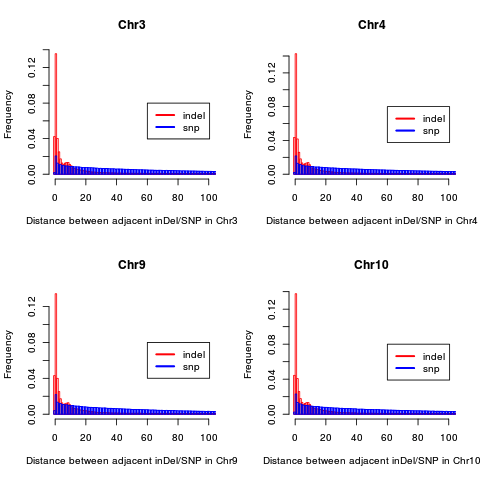

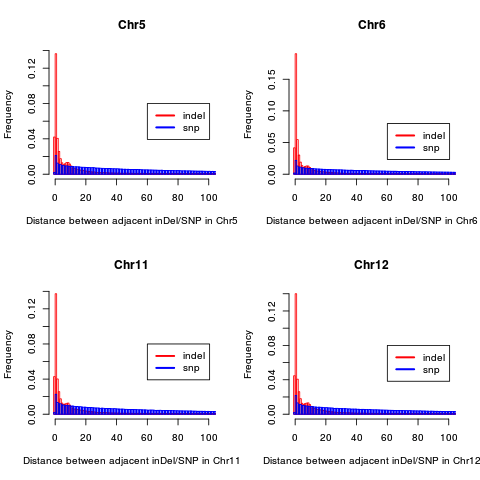

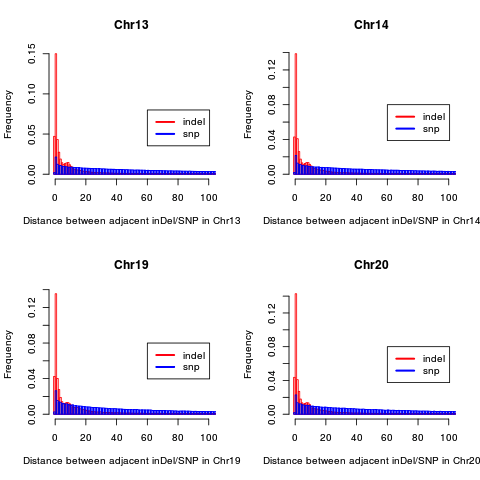

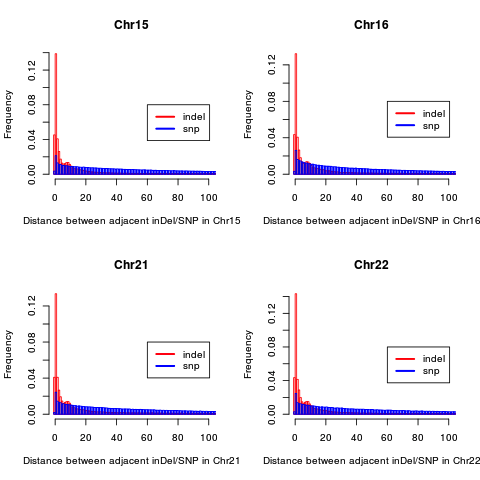

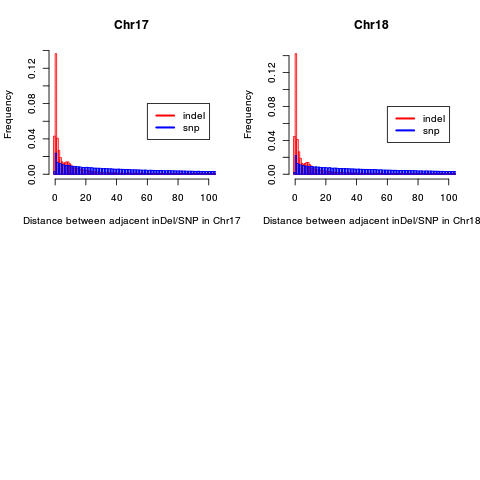


Figure 2. Histogram of adjacent SNP distribution for human chromosome 1-22

Figure 3. QQ-plot of adjacent-SNP distance distribution for human chromosome 1-22

Figure 4. Histogram of adjacent indel distribution (after redundancy filtration) for human chromosome 1-22

Figure 5. QQ-plot of adjacent-indel distance distribution (after redundancy filtration) for human chromosome 1 to 22
